# Medical History from the Earliest Times

**Published:** 1893-07-29

**Authors:** E. T. Withington


### Medical History from the Earliest Times.

By E. T. Withington, M.A., M.B. Oxon.

### LXIV.—THE ORIGIN OF MODERN MEDICINE.

When Lancisi found the anatomical drawings of Eustachius, which had been hidden away at Rome for 150 years, he sent them to a young friend of his, then professor of theoretic medicine at Padua, requesting him to point out what discoveries they contained which had been rediscovered during that interval. The answer, which was returned within eight days, was published with the plates in 1714, and so increased its author's reputation, already established by certain "Adversaria anatomica," that he received what might be called the blue riband of the medical professorate, the chair of anatomy at Padua, illustrious through the labours of Vesalius, Columbus, Fallopius, Fabricius, Gasserius, and Spigelius. It was now destined to receive a further accession of renown, for the name of the new professor was John Baptist Morgagni (1682-1771).

The great Eustachius regretted in his old age that he had not devoted himself to "that more obscure side of anatomy, that field so little cultivated yet so fertile in discoveries important to the healing art," the study of morbid structures, and this possibly determined the direction of Morgagni's work, resulting after forty-six years in the production of his famous "Seats and Causes of Disease, Investigated by Anatomy" (1661), one of the great books of medicine, which gained for its author the title of "Father of Pathology." Not that Morgagni was the first pathologist, any more than Hippocrates was the first physician, but he combined in himself all the knowledge of his predecessors in that department, while avoiding their errors. Thus his perfect acquaintance with ordinary anatomy prevented him from mistaking normal for abnormal structures, or *vice versa*, as the older pathologists nad often done. His predecessors had usually confined themselves to noticing extraordinaiy cases, but Morgagni, like Haller, held that the commonest diseases are the most important, and demand the most careful investigation. Above all, he was not content to notice the morbid changes only, but sought in every case to connect them with the history of the patient's disorder, thus avoiding the error—not entirely unknown among his successors—of confounding pathology with morbid anatomy, and diseases with organic lesions. He never even uses the term "pathological anatomy," so common since his day, for to him anatomy was the means, not the end of his work, which was to investigate the nature of disease. His eagerness to trace the connection between function and structure naturally led him to vivisections, and we find him opening the body of a living animal, "more majorum," as he calls it, to find out whether the pericardium is closely applied to the heart during life. To attempt to give an idea of Morgagni's great work by a few quotations would be like sampling a house by a basket of bricks, and it must suffice here to say that since the publication of the "Seats and Causes" the foremost question with a physician when examining a patient has been Where is the disease?

By a curious coincidence there appeared in the same year as Morgagni's two large folios a small pamphlet by Joseph Leopold Auenbrugger, a junior physician at the Vienna Hospital, which was destined to give important aid in answering the above question. This work, entitled "A New Invention for Discovering Obscure Thoracic Diseases by Percussion of the Chest," contained the results of six years' experience, and gave a wonderfully complete account of the sounds got by percussing various parts of the chest in health and disease, their value in diagnosis and prognosis, and the diseases which can and which cannot be so distinguished. Even the fremitus and relative mobility of the chest wall are not unnoticed, and one was astonished that Auenbrugger should have failed to complete his work by the discovery of auscultation. His clinical results were confirmed by post mortem examinations, and contemporary writers admit that the method enabled him to treat cases of pleurisy and empyæma with remarkable success; yet the importance of the "new invention" was recognised only by a minority till, in 1808, Napoleon's physician, Corvisart, published a French translation, with a commentary and preface which did equal honour to himself and the author. Since then the prayer with which Auenbrugger concludes his work has been abundantly answered: "Cedant haec miseris ægris in solatium, veris autem medicinæ cultoribus in incrementum artis. Quod opto."

The discovery became doubly valuable on the publication of the treatise, "On Mediate Auscultation," by R. T. H. Laennec, in 1819, about four years after he had invented the stethoscope. Direct auscultation was, as we have seen, not unknown to Hippocrates, and had been practised to some slight extent by his successors; but Laennec may claim the honour of discovering a more convenient method, of introducing it into general use, and of establishing its great importance in practice. We need scarcely, however, dilate upon this, for has not the stethoscope become a sort of crest or badge of the modern physician?

Less striking, but no less valuable in its results, was the work of M. F. X. Bichat, the shortest lived but one of the greatest of the heroes of medicine. Only one side of this work can be noticed here, his foundation of a new science now called histology, but then known as general anatomy. Bichat decomposed the organs of the body into their constituent elements, showing that they are made up of tissues, each possessing definite physical and vital properties, and he declared, as Boerhaave and Haller had done before him, that it is the physician's business not to speculate about abstract principles, but to trace the connection between the phenomena of life and the properties of the tissues in which they are manifested. He further pointed out that pathological phenomena are due to an alteration in these properties, and that the object of therapeutics is to restore, as far as possible, the natural type. To use his own language: "Le rapport des propriétés comme causes avec les phenomènes comme effets est un axiome presque fastidieux à répeter aujourdhui en physique, en chemie, et en astronomie. Si mon ouvrage établit un axiome analogue dans les sciences physiologiques il aura rempli son but." He died in his 31st year, July 22nd, 1802, from a malady acquired in the dissecting room. The next day Corvisart wrote to Napoleon: "Bichat has just fallen on a battlefield which numbers more than one victim. No one has done so much and so well in so short a time."

The three processes here briefly sketched, local pathology, local diagnosis, and the study of the tissues, form as it were, the three legs of the tripod, upon which the genius of modern medicine took her seat. There she received inspiration from many and varied sources; so many and so varied, indeed, that they cannot be adequately discussed within the limits of this short outline. We have followed the healing art in its long wanderings of something like forty centuries, and brought it at last to the borders of a land of promise. The occupation of this scientific Canaan may be said to have begun with the present century, and a magnificent specimen of its products had already been obtained in the great discovery of vaccination, the first fruits, we may fairly hope, of a vintage to be gathered in our own generation. But modern medicine differs so widely from that which preceded it, both in principles and complexity, that their point of junction forms a convenient termination for the present history, a main object of which has been to show that there were heroes before Agamemnon, and great physicians before the invention of the stethoscope. The writer has also attempted to point out the close connexion which exists between the history of medicine and that of general civilisation. The healing art is not, as some seem to think, the product of a special class, upon whose mistakes and failures the rest of mankind may look down with amusement or contempt; but is rather the final outcome of the efforts of the human race in all ages, to solve the perpetual riddles of a sphinx, who punishes every error without passion, but without pity. And if wild guesses at a solution have sometimes been made, it has usually been with the not ignoble object of seizing upon some general principle or panacea, which might forstall the slow progress of science, and afford some immediate relief to the innumerable bodily ills of man. Nor have the wildest guesses been entirely worthless. In the long pilgrimage up the hill difficulty some have stumbled on the dark mountains of error on the one side, and some have lost themselves in the great wood of vague speculation on the other, but on the whole there has been an almost continuous progress, a progress assisted not only by the labours of such men as Harvey and Bichat, but even by the ill-directed energies of a Paracelsus, a Brown, or a Hahnemann.

